# Role of elastography strain ratio and TIRADS score in predicting malignant thyroid nodule

**DOI:** 10.20945/2359-3997000000328

**Published:** 2021-02-24

Where you read (AUTHORS):

**Hussein Hassan Okasha** ^1^

https://orcid.org/0000-0002-0815-1394

**Mona Mansor** ^1^

https://orcid.org/0000-0002-7274-5429

**Nermine Sheriba** ^2^

https://orcid.org/0000-0002-1222-8948

**Maha Assem** ^1^

https://orcid.org/0000-0003-3295-2586

**Yasmine Abdelfattah** ^1^

https://orcid.org/0000-0002-2465-3757

**Omar A. Ashoush** ^3^

https://orcid.org/0000-0003-3992-9856

**Maha Rakha** ^3^

https://orcid.org/0000-0002-8287-7599

**Dalia Abdelfattah** ^4^

https://orcid.org/0000-0002-8598-7128

**Shereen Sadik El-Sawy** ^2^

https://orcid.org/0000-0001-9642-4657

**Mai Elshenoufy** ^1^

https://orcid.org/0000-0002-9781-9463

**Ahmed Amr Mohsen** ^5^

https://orcid.org/0000-0003-3893-1524

**Heba Kamal Sedrak** ^1^

https://orcid.org/0000-0001-9143-3247

**Abeer Awad Abdellatif** ^1^

https://orcid.org/0000-0001-9945-9767

^1^ Kasr Al-Aini Hospitals, Cairo University, Cairo, Egypt.

^2^ Ain Shams University, Cairo, Egypt.

^3^ Kasr Alainy Faculty of Medicine, Cairo University, Cairo, Egypt.

^4^ Biostatistic and Cancer Epidemiology Department, National Cancer Institute, Cairo University, Cairo, Egypt.

^5^ National Research Centre, Cairo, Egypt.

Should read:

**Hussein Hassan Okasha** ^1^

https://orcid.org/0000-0002-0815-1394

**Mona Mansor** ^1^

https://orcid.org/0000-0002-7274-5429

**Nermine Sheriba** ^2^

https://orcid.org/0000-0002-1222-8948

**Maha Assem** ^1^

https://orcid.org/0000-0003-3295-2586

**Yasmine Abdelfattah** ^1^

https://orcid.org/0000-0002-2465-3757

**Omar A. Ashoush** ^1^

https://orcid.org/0000-0003-3992-9856

**Maha Rakha** ^1^

https://orcid.org/0000-0002-8287-7599

**Dalia Abdelfattah** ^3^

https://orcid.org/0000-0002-8598-7128

**Shereen Sadik El-Sawy** ^1^

https://orcid.org/0000-0001-9642-4657

**Mai Elshenoufy** ^1^

https://orcid.org/0000-0002-9781-9463

**Ahmed Amr Mohsen** ^4^

https://orcid.org/0000-0003-3893-1524

**Heba Kamal Sedrak** ^1^

https://orcid.org/0000-0001-9143-3247

**Abeer Awad Abdellatif** ^1^

https://orcid.org/0000-0001-9945-9767

^1^Kasr Al-Aini Hospitals, Cairo University, Cairo, Egypt.

^2^Ain Shams University, Cairo, Egypt.

^3^Biostatistic and Cancer Epidemiology Department, National Cancer Institute, Cairo University, Cairo, Egypt.

^4^ National Research Centre, Cairo, Egypt.

